# Clinical applications of urinary sodium in heart failure from prognostic marker to clinical tool

**DOI:** 10.1002/ehf2.15350

**Published:** 2025-06-25

**Authors:** Jeroen Dauw

**Affiliations:** ^1^ Cardiovascular Center Aalst AZORG Hospital Aalst Belgium

Sodium plays a central role in the pathophysiology of heart failure, where its retention drives congestion and volume overload.^1^ Urinary sodium (UNa) provides direct insight into renal sodium handling and reflects the combined influence of neurohormonal activation, renal perfusion and tubular function; all of which are central to the syndrome. Although UNa has only recently entered clinical heart failure care, it is gaining recognition as a prognostic marker, as shown in the current analysis, and is increasingly considered for therapeutic and monitoring applications.^2^, ^3^

In this issue, a post‐hoc analysis of the Efficacy of Saline Hypertonic Therapy in Ambulatory Patients with HF (SALT‐HF) trial examines the association between early natriuretic response and 30‐day clinical outcomes in ambulatory patients with worsening heart failure.^4^ SALT‐HF was a randomized trial investigating the effect of adding hypertonic saline to intravenous loop diuretics in this population.^5^, ^6^ The authors evaluated UNa and urine output collected over 3 h following intravenous loop diuretic administration and observed that low UNa, but not low urine volume, was independently associated with higher risk of adverse outcomes, including death, hospitalization, or need for repeat IV diuretics. These results reinforce the idea that natriuresis may reflect more meaningful decongestion than diuresis alone, in terms of both alignment with underlying pathophysiology and its association with outcomes.

However, several considerations temper the interpretation of these findings. Most importantly, 30‐day outcomes are influenced by multiple factors beyond the initial response to therapy. The impact of treatment adjustments, outpatient follow‐up and changes in disease trajectory likely play a significant role and were not fully captured. Furthermore, the inclusion of hypertonic saline in half of the population may have influenced natriuretic response, and no subgroup analysis according to treatment arm was performed. Finally, although three‐hour urine collections are pragmatic, they may not reflect the full duration of loop diuretic action, which typically peaks between 4 and 6 h.

From a mechanistic perspective, these findings support the concept that sodium retention is the primary driver of congestion in heart failure, with fluid accumulation occurring as a secondary consequence. This underlines the relevance of targeting natriuresis rather than fluid loss. In a subanalysis of the Renal Optimization Strategies Evaluation in Acute Heart Failure (ROSE‐AHF) study, patients with low urinary sodium excretion had worse outcomes even when achieving a negative fluid balance,^7^ suggesting that UNa more directly reflects the pathophysiological processes underlying congestion and may be the best target for decongestion.

Although the SALT‐HF analysis was observational, it aligns with prior data showing that early natriuresis is a meaningful prognostic marker both as a single sample value as when assessed serially during decongestion.^3^, ^8^ More importantly, there is the growing body of evidence suggesting that UNa may serve not only as an indicator but also as a modifiable parameter in treatment decision‐making.^2^ The use of UNa to guide diuretic response is already supported by the 2021 ESC heart failure guidelines.^9^ Furthermore, interventional studies such as the Efficacy of a Standardized Diuretic Protocol in Acute Heart Failure (ENACT‐HF) study^10^, ^11^ and Pragmatic Urinary Sodium‐based AlgoritHm in Acute Heart Failure (PUSH‐AHF) study^12^ have shown that structured diuretic protocols guided by early UNa response lead to more effective and timely decongestion. While these trials did not demonstrate improvements in hard endpoints, they proved that UNa‐guided therapy is feasible and safe and leads to meaningful changes in management. This concept was extended in the Readily Available Urinary Sodium Analysis in Patients with Acute Decompensated Heart Failure (EASY‐HF) trial, which applied a nurse‐led diuretic algorithm based on UNa in the acute hospital setting, improving congestion scores through standardized, protocolised care.^13^

Another area of interest has been the use of UNa as an outpatient monitoring tool to detect congestion early. As neurohormonal activation directly impairs renal sodium excretion, a drop in UNa might be expected early before clinical signs occur. In an observational study, morning UNa sodium remained stable over 30 weeks, but a drop was noticed 1 week before an acute heart failure episode occurred.^14^ In addition, the Readily Available Urinary Sodium Analysis to Stop Loop Diuretics in Patients with Heart Failure (EASY‐STOP) study evaluated UNa in the ambulatory setting to monitor patients after diuretic withdrawal.^15^ A failure to increase first void UNa after reducing or stopping diuretics appeared to identify patients who did not tolerate diuretic withdrawal. This suggests a potential role for UNa, with a drop of UNa as an early warning signal in chronic heart failure management. Although outpatient monitoring is an appealing application of UNa, interventional studies are needed to determine its exact role and value in clinical practice.

*Figure* 1 summarizes the potential clinical applications of UNa in heart failure. UNa can be used as a prognostic marker to stratify risk, as a therapeutic parameter to assess diuretic response and adjust therapy and is being explored as a potential tool to detect early signs of decompensation in ambulatory follow‐up.

**Figure 1:**
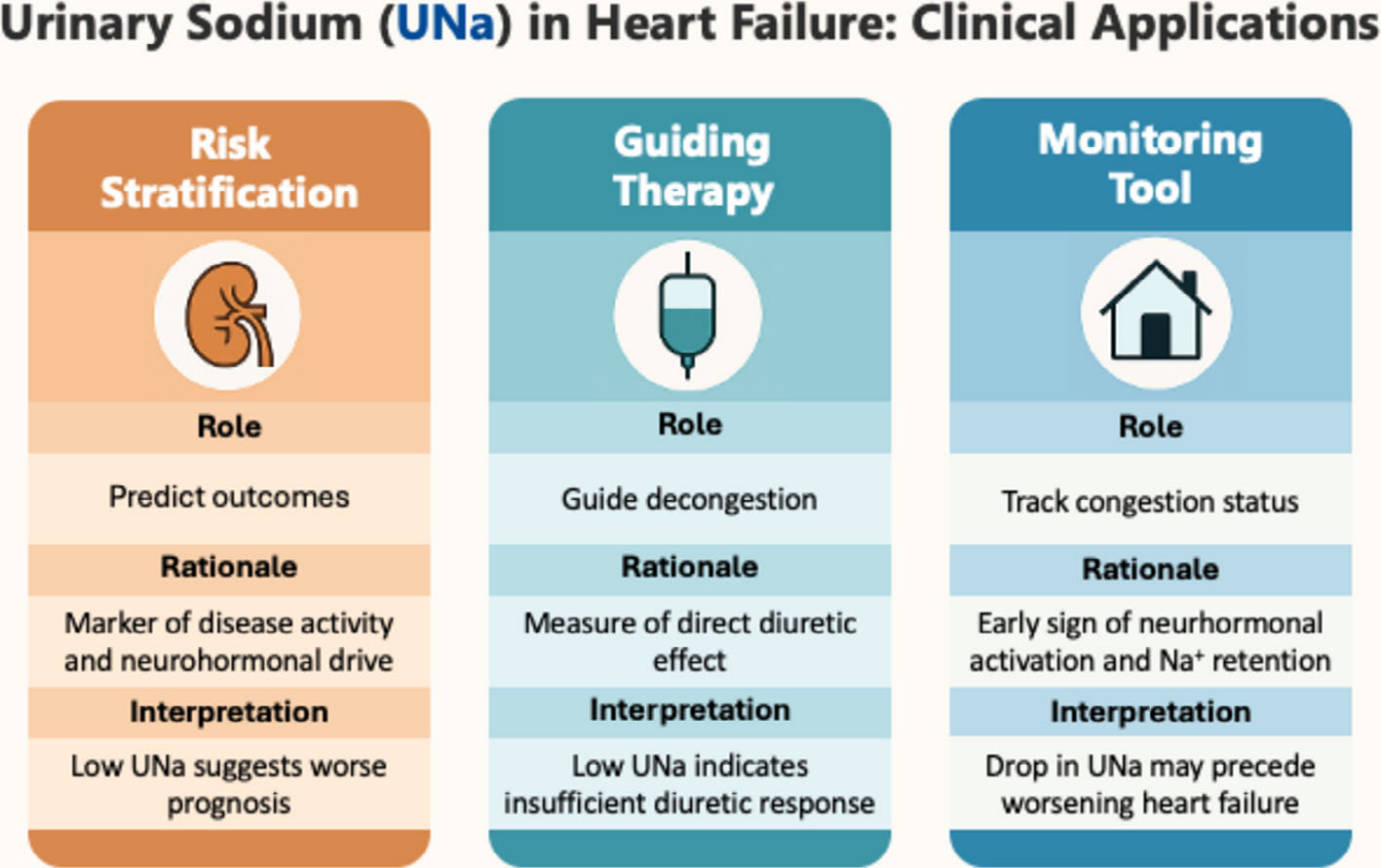
Clinical roles and rationale for urinary sodium (UNa) in heart failure UNa plays a clinically relevant role in three domains: as a prognostic marker for risk stratification, as a therapeutic parameter to guide diuretic response, and as a potential monitoring tool to detect early decompensation. These uses are grounded in the physiological link between sodium retention and congestion in heart failure. While its prognostic and therapeutic roles are supported by growing evidence, the use of UNa for ambulatory monitoring remains exploratory. The figure outlines each role, its mechanistic rationale and interpretation in practice.

In summary, UNa offers a simple and clinically relevant parameter with both prognostic and therapeutic value. The SALT‐HF data confirm its prognostic significance and support further integration into hospital‐based diuretic protocols and post‐discharge care strategies. Although the study did not evaluate treatment guidance directly, the results strengthen the idea that UNa serves as a practical tool to connect underlying pathophysiology with real‐time clinical decision‐making.

## Funding

No funding was provided for this editorial.

## Conflict of interest

JD received speaker fees from AstraZeneca, Bayer, Boehringer‐Ingelheim, and Novartis and travel grants from AstraZeneca, Bayer and Daiichi Sankyo.
